# Burden and Trend of Macrosomia and Large-for-Gestational-Age Neonates Attributable to High Pre-Pregnancy Body Mass Index in China, 2013–2017: A Population-Based Retrospective Cohort Study

**DOI:** 10.3390/healthcare11030331

**Published:** 2023-01-22

**Authors:** Shuai Zeng, Ying Yang, Chunying Han, Rongwei Mu, Yuzhi Deng, Xinyi Lv, Wenlu Xie, Jiaxin Huang, Siyu Wu, Ya Zhang, Hongguang Zhang, Yuan He, Zuoqi Peng, Yuanyuan Wang, Haiping Shen, Qiaomei Wang, Yiping Zhang, Donghai Yan, Long Wang, Xu Ma

**Affiliations:** 1 Institute of Epidemiology and Statistics, School of Public Health, Lanzhou University, Lanzhou 730000, China; 2 National Research Institute for Family Planning, Beijing 100081, China; 3 National Human Genetic Resources Centre, Beijing 100035, China; 4 Graduate School of Peking Union Medical College, Beijing 100730, China; 5 Department of Maternal and Child Health, National Health Commission of the PRC, Beijing 100044, China

**Keywords:** fetal macrosomia, large-for-gestational-age, body mass index, population attributable fraction, cohort studies

## Abstract

**Background:** The world is transitioning to an obese future, but few studies have measured the burden of increased maternal body mass index (BMI) on pathological fetal overgrowth, especially the trends in this burden and its heterogeneity in populations with different characteristics. **Methods:** A population-based retrospective cohort study was conducted with 7,998,620 Chinese females who had participated in the National Free Pre-Pregnancy Check-ups Project and became pregnant during 2013–2017. The proportions of macrosomic and LGA neonates attributable to high BMI (population attributable fraction, PAF) and annual percent change of yearly PAFs were estimated. **Results:** We found that the burden of macrosomic and LGA (large-for-gestational-age) neonates attributable to high pre-pregnancy BMI increased among Chinese females with planned pregnancies during 2013–2017. The PAF of macrosomia attributable to high BMI increased from 3.16% (95% confidence interval: 2.97–3.35%) to 7.11% (6.79–7.42%) by 23.60% (16.76–30.85%) annually, and the PAF of LGA increased from 2.35% (2.21–2.48%) to 5.00% (4.79–5.21%) by 21.98% (16.14–28.11%) annually. Our study identified that participants with disadvantaged socioeconomic status (including those without higher education, living in provinces with GDP per capita < 40,000 CNY, tier IV, and tier V cities) and residing in northern and southwestern China were at high risk of a rapidly expanding burden. **Conclusions:** Government authorities should control pre-pregnancy BMI through nationwide intervention programs and direct more resources to focus on the unfair burden on females with disadvantaged socioeconomic status.

## 1. Introduction

Pathological fetal overgrowth, including macrosomia and large-for-gestational-age (LGA), is a clinically important reproductive health problem and has long-lasting metabolic impacts on the next generation [1,2,3]. The prevalence of macrosomia and LGA is approximately 1–20% and 4–22% worldwide and 3–14% and 7–22% in China, respectively [4,5,6,7]. The risk of macrosomia and LGA in neonates could be effectively reduced by blood sugar management, exercise, weight gain management, and lifestyle intervention before conception and during gestation [8].

Established evidence has concluded a clear causal relationship between maternal high pre-pregnancy body mass index (BMI) and the risk of increased birth weight; maintaining a healthy pre-pregnancy BMI level could significantly reduce the risk of macrosomia and LGA in offspring [9]. The world is still transitioning to an obese future and high BMI is clearly not well controlled worldwide [10]. In China, overweight and obesity defined by WHO BMI cut-offs in adult women have increased dramatically from 24.6% and 3.7% in 2004 to 36.7% and 7.2% in 2018, respectively, making a growing number of reproductive-aged women at risk of giving birth to macrosomic and LGA neonates [11]. Additionally, high pre-pregnancy BMIs are found to play a crucial role in the epidemic of metabolic diseases in the next generation, burdening global public health [2,3]. Compared with increasing numbers of studies on high pre-pregnancy BMI and the risk of birth weight and LGA, only a few international studies have assessed the trend and burden of macrosomia and LGA attributable to high pre-pregnancy BMI. Especially in China, a country with a rapidly rising high BMI problem, there is a lack of sufficient evidence to guide public health practice.

In this retrospective cohort study, we aimed to characterize the trend and burden of macrosomic and LGA neonates attributable to high pre-pregnancy BMI in mainland China during 2013–2017.

## 2. Materials and Methods

### 2.1. Study Setting

The current study is based on data from the National Free Pre-Pregnancy Check-ups Project (NFPCP) in China, which was launched in pilot counties by the National Health Commission and the Ministry of Finance in 2010. The NFPCP is intended to provide free pre-pregnancy health examination and counselling services for couples planning to become pregnant in the next six months. The NFPCP became nationally representative after the project expanded to all counties in 2013.

In brief, NFPCP includes preconception examination, early pregnancy follow-up, and outcome follow-up. At the preconception examination, a face-to-face structured questionnaire, physical examination, and laboratory tests were applied by trained healthcare workers to obtain the baseline characteristics of the participants. After the completion of the preconception examination, participants will be followed up via telephone at trimester intervals to determine whether they have successfully conceived. Once pregnancy events are recorded, participants will be asked to undergo ultrasonography to confirm the conception. Pregnancy participants will be recontacted for pregnancy outcomes within 1 year of the completion of early-pregnancy follow-up. Detailed design, implementation, and quality control can be found elsewhere [12].

### 2.2. Study Population

**Figure 1:**
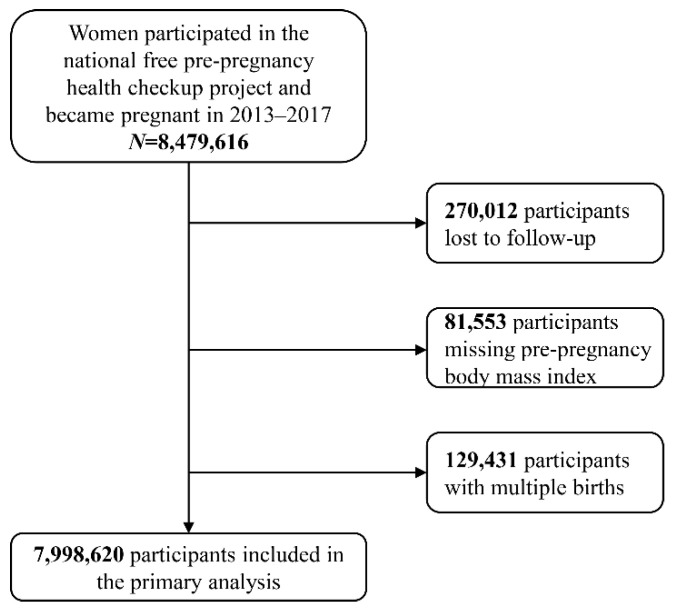
Flow chart for included participants.

In the current study, a total of 8,479,616 NFPCP participants who became pregnant from January 2013 to December 2017 were included. After excluding participants who were lost to follow-up, with missing BMI, and multiple births, 7,998,620 participants were included in the final analysis (Figure 1).

### 2.3. Exposure and Outcomes

Maternal high pre-pregnancy BMI is regarded as the main exposure, defined as the pre-pregnancy weight divided by the square of the height (kilograms/square meters) ≥ 25.0 kg/m^2^ using World Health Organization (WHO) criteria [13]. Height and weight measurements were performed indoors, with participants barefoot and wearing light clothing, and were measured using instruments that met national standards.

Both macrosomia and LGA are regarded as the main outcomes. Macrosomia is defined as a birth weight of >4000 g, and LGA is determined by having a birth weight greater than the 90th percentile for all NFPCP deliveries of the same sex and gestational age [14,15].

### 2.4. Covariates

Covariates used in the current study include maternal and paternal age (20–24, 25–29, 30–34, and ≥35 years), higher education (education ≥ 10 years, categorized into no and yes), nationality (Han and minority (including the remaining 55 ethnic groups), the ethnic Han are the ethnic majority in China, while the ethnic minority account for less than 9% of the population, household registration (rural and urban), smoking (no and yes), secondhand smoking (no, 1–14, and ≥ 15 min/day), alcohol drinking (no and yes), parity (nulliparous and parous), infant sex (male and female), gross domestic product (GDP) per capita (<40,000, 40,000–, 50,000–, and 70,000– CNY) [16], city tier (tier I (4 cities), new tier I (15 cities), tier II (30 cities), tier III (70 cities), tier IV (90 cities), and tier V (128 cities)), and region (northeast, north, northwest, east, central, south, and southwest). The city tier is based on the standard classification of the official Chinese media, a comprehensive classification system that reflects the concentration of resources, political and economic development, lifestyle diversity, and future development prospects of cities in mainland China [17].

Only maternal age, paternal age, higher education, nationality, household registration, smoking, secondhand smoking, alcohol consumption, parity, infant sex, and region were considered as confounders in multivariate analyses. Because the substantial influence of infant sex on the outcomes and levels of service may vary across the entire nation, we also additionally adjusted for infant sex and the region of provinces.

### 2.5. Statistical Analysis

We used numbers (*n*) and percentages (%) to describe the baseline characteristics of participants, and we applied χ^2^ tests to determine differences in baseline characteristics across the last menstrual period (LMP) years.

Yearly multivariate-adjusted relative risks (RRs), along with the prevalence of higher BMI, incidence of macrosomia and LGA were calculated. RRs and their 95% corresponding confident intervals (CIs) were estimated using a log-binomial regression model with the adjustment of maternal age, paternal age, higher education, nationality, household registration, smoking, secondhand smoking, alcohol consumption, parity, infant sex, and region. We used the normal weight defined by WHO criteria (18.5–24.9 kg/m^2^) as the reference group when estimating RRs.

The population attributable fraction (PAF) was used to measure the burden of macrosomia and LGA attributable to high BMI among the study population. Both crude and confounders-adjusted PAFs and their 95% CIs were calculated. The corresponding formulas are listed below:

$$Crude\;PAF=\frac{p\;\left(RR-1\right)}{1+p\;\left(RR-1\right)}\;Confounders-adjusted\;PAF=pd\;\left(\frac{RR-1}{RR}\right)$$

where *p* is the proportion of the population exposed to high BMI, *pd* is the proportion of macrosomic or LGA neonates exposed to higher BMI, *RR* is the crude RR for crude PAF and multivariate-adjusted RR for confounders-adjusted PAF [18].

We used annual percent change (APC), estimated using a log-linear model, to measure the temporal trends of indices, including prevalence of high BMI, incidences of macrosomia and LGA, RRs of macrosomia and LGA with high BMI, PAFs of macrosomia and LGA attributable to high BMI.

We used subgroup analyses based on baseline characteristics to explore the within-population heterogeneity of burden and trends of macrosomia and LGA attributable to high BMI, and simple linear regression was used to fit trends in PAF and APC for each subgroup.

Statistical analyses were performed using R (version 4.0.4). Graphs were plotted using GraphPad Prism (version 9.4.0). A two-sided *p* value of <0.05 was considered statistically significant.

## 3. Results

The baseline characteristics of the participants by LMP years are presented in Table 1. The proportions of participants with maternal age ≥30 years (17.17% to 32.51%), paternal age ≥30 years (28.71% to 43.78%), higher education (33.80% to 42.14%), and urban residency (6.13% to 12.93%) increased during 2013–2017, and the proportions of participants who were nullipara (70.51% to 49.24%) decreased. Most participants did not smoke or drink alcohol daily and were not exposed to secondhand smoke.

The prevalence of high BMI, incidence of macrosomia and LGA, and RRs of macrosomia and LGA with high BMI significantly changed during the study period (Table 2). The prevalence of high BMI increased from 8.78% in 2013 to 12.91% in 2017, with an APC of 11.56% (5.36–18.13%). The incidence of macrosomia and LGA decreased from 4.79% and 9.31% to 3.32% and 8.08%, respectively, with annual declines of 7.89% (3.15–12.40%) and 3.15% (1.26–5.01%), respectively. RRs of macrosomia and LGA with high BMI increased from 1.34 (1.31–1.37) and 1.25 (1.23–1.27) in 2013 to 1.54 (1.50–1.58) and 1.37 (1.35–1.39) in 2017 by 3.51% (2.39–4.64%) and 2.29% (1.74–2.84%) annually, respectively (*p* for trend < 0.01).

Table 3 describes PAFs of macrosomia and LGA attributable to high BMI from 2013 to 2017. After adjustment for potential confounders, we found that the PAF of macrosomia attributable to high BMI increased from 3.16% (2.97–3.35%) in 2013 to 7.11% (6.79–7.42%) in 2017 by 23.60% (16.76–30.85%) annually (*p* for trend < 0.01), and the PAF of LGA increased from 2.35% (2.21–2.48%) to 5.00% (4.79–5.21%) by 21.98% (16.14–28.11%) annually (*p* for trend < 0.01). Sensitivity analyses after excluding participants with pre-pregnancy diabetes, hypertension or thyroid disease showed consistent results (Tables S1–S3).

**Figure 2:**
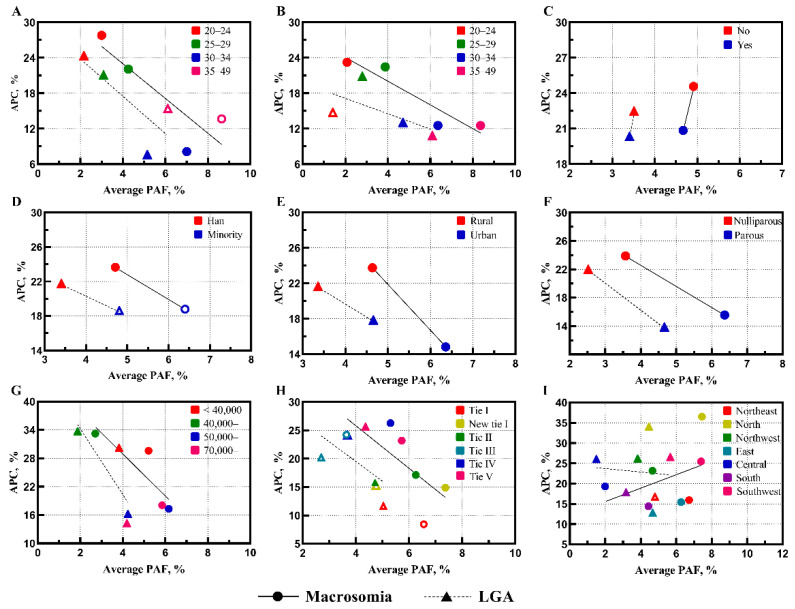
Within-Population Heterogenicity of Burden and Trends of Macrosomic and LGA Neonates Attributable to High Pre-pregnancy Body Mass Index. Legend: (**A**) = maternal age; (**B**) = paternal age; (**C**) = higher education; (**D**) = nationality; (**E**) = household registration; (**F**) = parity; (**G**) = GDP per capita; (**H**) = city tier; and (**I**) = region. The straight and dashed lines are the linear approximation of macrosomia and LGA, respectively. Hollow symbols represent annual percent change of nonstatistical significance (*p* > 0.05).

Consistent results can be found in subgroup analyses (Figure 2). In general, subgroups with higher PAFs have lower APCs, and vice versa. The two subgroups of higher education and region (only in macrosomia) have reversed trends. Macrosomic and LGA neonates attributable to high BMI among participants without higher education were comparable to participants with higher education but had a considerably higher growth rate (APCs: 24.58% vs. 20.86% for macrosomia and 22.47% vs. 20.32% for LGA neonates). Participants living in provinces with GDP per capita < 40,000 CNY have a relatively higher burden and rapid growth of macrosomia (PAF: 5.21%; APC: 29.60%) and LGA neonates (PAF: 3.83%; APC: 30.20%) attributable to higher BMI. A similar situation could be observed in participants living in tier IV and tier V cities. In terms of subgroup analyses according to region, participants in northern China and southwestern China have remarkable expansion of macrosomic (PAF: 7.46% and 7.41%; APC: 36.54% and 25.49%) and LGA neonates (PAF: 4.46% and 5.67%; APC: 34.04% and 26.51%) attributable to higher BMI.

## 4. Discussion

In the current population-based retrospective cohort study of 7,998,620 planned pregnancies in mainland China, we measured the burden of macrosomic and LGA neonates attributable to high pre-pregnancy BMI during 2013–2017, and for the first time identified an increasing trend in this burden, as well as identifying populations at high risk of the burden. Our findings provide solid support for public health practitioners and policy-makers to employ targeted strategies and interventions on reproduction health equity promotion.

Trends of macrosomic and LGA neonates attributable to high pre-pregnancy BMI are easily neglected problems in public health practice. As the prevalence of high BMI is still climbing steadily worldwide, the growing body of macrosomic and LGA neonates attributable to high BMI is not surprising. Despite the decreased prevalence of macrosomia and LGA, trends of macrosomic and LGA neonates attributable to high BMI outpaced the increasing rate of high BMI. Additionally, compared to 23.4% of macrosomic and 9.0% of LGA neonates in Australia during 2010–2014, 24.3% of macrosomia in Nova Scotia in Canada during 2004–2010, 13.7% of LGA in Amsterdam during 2003–2004, 24.87% of macrosomia and 17.36% of LGA (re-estimated for comparison) in Japan during 2010–2019, 13.18% of macrosomia(re-estimated for comparison) in Korea during 2013–2017, and 13.08% of LGA(re-estimated for comparison) in Korea during 2007–2009, the burdens of macrosomia (4.44%) and LGA (3.33%) attributable to high BMI were lower in the current population [19,20,21,22,23,24]. However, macrosomia and LGA attributable to high BMI in the current study had annual growth rates of 23.60% and 21.98%, respectively, which were far beyond 1.43% and 1.57% (recalculated for comparison) in Australia during 1990–2014 [19]. Alternatively, if we change the definition of high BMI to the Chinese domestic standard of ≥24.0 kg/m^2^, the growth rates were not substantially changed (Table S4). A growing burden of macrosomia and LGA attributable to high BMI in mainland China is expected in the coming decade, accelerating adverse impacts on the metabolic health of the next generation.

All the increased prevalence of high BMI, the decreased prevalence of macrosomic and LGA neonates, and the increased association magnitude of high BMI with the risk of macrosomia and LGA contributed to the growing burden. With increased exposure to the obesogenic environments of unhealthy dietary patterns and reduced physical activity, the increased trend of high BMI in the current population was identical to the trend in the general population [11,25,26]. However, the magnitude of growth greatly exceeded growth rates (3.55% during 2000–2018 and 1.05% during 2013–2018) recalculated using data from the China Chronic Disease and Risk Factors Surveillance (CCDRFS) [11]. Because of the younger age structure in NFPCP participants, the prevalence of high BMI was well below that of adult females (43.9%) in 2018 in mainland China [11]. In addition, the decreased prevalence of macrosomia and LGA could be mainly attributable to intensive antenatal care services for successful gestational weight gain control [27]. Control of modifiable risk factors for macrosomia and LGA during gestation would increase PAF attributed to pre-pregnancy modifiable risk factors, including BMI in the current study. We also acknowledged the lower RRs for macrosomia and LGA with pre-pregnancy BMI when compared to the results from the Western population, which could be attributed to the different BMI levels in two kinds of high BMI populations [19,20,28]. The reason could also be applied to explain the increased magnitude of RRs in the current study, where the mean BMI levels were increased in high BMI participants. When comparing the results with those of Japan and Korea, even using the same high BMI criterion (≥25 kg/m^2^), their high pre-pregnancy BMI ORs for macrosomia and LGA were more than 1.75 and 2.15 times higher than those of China, respectively, and it is speculated that the RR values in China have a tendency to further approach those of Japan, Korea, and Western countries, and further studies are needed for this phenomenon [23,24]**.**

It is also imperative to explore the within-population heterogeneity and identify high-risk populations to guide public health practitioners and policy-makers to appropriately allocate healthcare resources to lower the growing burden. In China, after decades of health legislation and investment in health resources, the government has constructed a system of free preconception screening, maternal and child health management, and newborn screening. Hospitals above the second level with sufficient obstetricians and maternal and child health institutions are primarily responsible for delivery, while primary institutions and community health services focus on prenatal care and high-risk pregnancy screening, making a significant contribution to improving maternal and newborn health [29,30]. However, inequalities in maternal health rights occur due to the vast size of China, large differences in economic, medical, and healthcare resources between regions, an inadequate health insurance system for the mobile population, and a lower density of maternal and child health workers in primary care facilities than internationally recommended [31]. The growing burden of macrosomic and LGA neonates attributable to high pre-pregnancy BMI consisted of nearly all subgroups, which implied universal insufficiency of high pre-pregnancy BMI prevention, and more public health resources should be invested in the field. Here, we also identified that participants without higher education and living in provinces with GDP per capita < 40,000 CNY, cities of tier IV and tier V, and northern and southwestern China were at high risk of a rapidly expanding burden of macrosomia and LGA attributable to high pre-pregnancy BMI. These identified high-risk populations potentially shared similar disadvantages in socioeconomics, including awareness of sufficient knowledge on pre-pregnancy health promotion, accessibility of qualified reproduction health services, and adequate antenatal care.

Our study also has significant public health implications. The nationwide growing burden calls for an urgent need to stay alert to metabolic pressure from the current generation and preventing high pre-pregnancy BMI. The central role of women with pregnancy intentions in transgenerational metabolic diseases among public health policy-makers and practitioners should be acknowledged. Practical ways include strengthening health counselling services focusing on healthy BMIs and adverse impacts of higher BMIs on the two generations in premarital check-ups, preconception check-ups, and fertility clinics [32,33]. The heterogeneity also highlights the inequitable growing burden in high-risk females with low socioeconomic status, and more healthcare resources should be mobilized to discontinue adverse transitions in socioeconomically disadvantaged communities.

In addition to using a retrospective cohort study based on a large sample size to measure the burden of macrosomic and LGA neonates attributable to high pre-pregnancy BMI in mainland China, our study has the advantage of identifying, for the first time, the rate and direction of change in this burden and the high-risk populations that are under threat in this variation, leading to better targeting of resources for policy.

Our study has some limitations. Firstly, our study has insufficient ability to measure central obesity-associated macrosomic and LGA neonates. Secondly, factors such as weight gain during pregnancy, reproductive assisted technology, and blood glucose have been reported to affect the birth weight of newborns [34,35,36]. However, the present study was unable to control for confounding factors, including maternal weight gain, infertility treatment, glycaemia control, macrosomia and LGA in previous pregnancies when estimating RRs, because relevant data were not collected. It would overestimate the burden of macrosomic and LGA neonates attributed to high pre-pregnancy BMI but has limited impacts on the trends of burden. The results in nulliparous participants and participants excluding pre-pregnancy diabetes, hypertension, or thyroid disease (Tables S1–S3) can alternatively assess the uncontrolled bias caused by these factors. Thirdly, unwanted pregnancies are not covered in the current study, which may lower the generalizability of our study. According to a survey published in 2013 of sexually active unmarried youth in Shanghai, China, less than 9.5% of unintended pregnancies continue, so we believe our results are still generalizable to the whole of pregnancies in mainland China [37]. Lastly, the study has limited power to determine long-term temporal trends of the burden, but the findings are still of public health value to shed light on high pre-pregnancy BMI control of the next decade.

## 5. Conclusions

In conclusion, the findings from this cohort study suggest that the growing body of high pre-pregnancy BMI should be comprehensively controlled and resources for reproductive health services should be directed towards socioeconomically disadvantaged communities to lessen the burden of macrosomic and LGA neonates.

## Figures and Tables

**Table 1:** Baseline characteristics of the included participants across LMP years.

Characteristics	2013 (*n* = 1,724,140)	2014 (*n* = 1,669,015)	2015 (*n* = 1,611,623)	2016 (*n* = 1,753,983)	2017 (*n* = 1,239,859)	*p*
Maternal Age, years						<0.001
20–24	657,608 (38.14)	556,039 (33.32)	482,719 (29.95)	382,674 (21.82)	268,003 (21.62)	
25–29	770,482 (44.69)	806,810 (48.34)	822,139 (51.01)	797,620 (45.47)	568,804 (45.88)	
30–34	233,108 (13.52)	234,462 (14.05)	231,734 (14.38)	361,927 (20.63)	273,983 (22.10)	
≥35	62,942 (3.65)	71,704 (4.30)	75,031 (4.66)	211,762 (12.07)	129,069 (10.41)	
Paternal Age, years						<0.001
20–24	382,952 (22.40)	310,199 (18.72)	260,441 (16.28)	191,515 (11.01)	139,167 (11.32)	
25–29	836,016 (48.89)	849,644 (51.28)	847,755 (52.98)	776,783 (44.65)	551,839 (44.90)	
30–34	353,500 (20.67)	350,423 (21.15)	346,579 (21.66)	448,080 (25.76)	338,981 (27.58)	
≥35	137,437 (8.04)	146,568 (8.85)	145,423 (9.09)	323,215 (18.58)	199,136 (16.20)	
Missing	382,952 (22.40)	310,199 (18.72)	260,441 (16.28)	191,515 (11.01)	139,167 (11.32)	
Higher Education						<0.001
No	1,095,682 (63.55)	1,017,974 (60.99)	897,629 (55.70)	955,085 (54.45)	652,205 (52.60)	
Yes	582,745 (33.80)	601,619 (36.05)	651,372 (40.42)	721,426 (41.13)	522,423 (42.14)	
Missing	45,713 (2.65)	49,422 (2.96)	62,622 (3.89)	77,472 (4.42)	65,231 (5.26)	
Nationality						<0.001
Han	1,578,694 (91.56)	1,525,842 (91.42)	1,473,028 (91.40)	1,607,399 (91.64)	1,128,913 (91.05)	
Minority	122,942 (7.13)	121,164 (7.26)	115,491 (7.17)	123,645 (7.05)	91,925 (7.41)	
Missing	22,504 (1.31)	22,009 (1.32)	23,104 (1.43)	22,939 (1.31)	19,021 (1.53)	
Household Registration						<0.001
Rural	1,618,453 (93.87)	1,544,734 (92.55)	1,451,580 (90.07)	1,537,184 (87.64)	1,078,553 (86.99)	
Urban	105,675 (6.13)	124,274 (7.45)	160,013 (9.93)	216,293 (12.33)	160,366 (12.93)	
Missing	12 (0.00)	7 (0.00)	30 (0.00)	506 (0.03)	940 (0.08)	
Smoking						<0.001
No	1,713,054 (99.36)	1,659,213 (99.41)	1,602,495 (99.43)	1,743,897 (99.42)	1,233,200 (99.46)	
Yes	4342 (0.25)	3221 (0.19)	3087 (0.19)	3408 (0.19)	2738 (0.22)	
Missing	6744 (0.39)	6581 (0.39)	6041 (0.37)	6678 (0.38)	3921 (0.32)	
Secondhand smoking, min/day						<0.001
No	1,624,386 (94.21)	1,597,278 (95.70)	1,551,638 (96.28)	1,689,288 (96.31)	1,194,750 (96.36)	
1–14	60,250 (3.49)	42,586 (2.55)	34,271 (2.13)	37,916 (2.16)	26,129 (2.11)	
≥ 15	32,971 (1.91)	22,311 (1.34)	19,573 (1.21)	20,042 (1.14)	15,118 (1.22)	
Missing	6533 (0.38)	6840 (0.41)	6141 (0.38)	6737 (0.38)	3862 (0.31)	
Alcohol Consumption						<0.001
No	1,666,488 (96.66)	1,615,724 (96.81)	1,560,839 (96.85)	1,698,200 (96.82)	1,201,907 (96.94)	
Yes	14,748 (0.86)	10,486 (0.63)	10,208 (0.63)	11,117 (0.63)	8575 (0.69)	
Missing	42,904 (2.49)	42,805 (2.56)	40,576 (2.52)	44,666 (2.55)	29,377 (2.37)	
Parity						<0.001
Nulliparous	1,215,682 (70.51)	1,108,008 (66.39)	1,045,340 (64.86)	826,378 (47.11)	610,468 (49.24)	
Parous	502,904 (29.17)	555,377 (33.28)	561,148 (34.82)	922,259 (52.58)	626,471 (50.53)	
Missing	5554 (0.32)	5630 (0.34)	5135 (0.32)	5346 (0.30)	2920 (0.24)	
Infant sex						
Male	865,840 (50.22)	832,099 (49.86)	801,885 (49.76)	870,408 (49.62)	636,012 (51.30)	
Female	784,004 (45.47)	765,036 (45.84)	742,513 (46.07)	810,032 (46.18)	585,914 (47.26)	
Missing	74,296 (4.31)	71,800 (4.31)	67,222 (4.17)	73,543 (4.19)	17,933 (1.45)	
GDP per Capita, CNY						<0.001
< 40,000	344,350 (19.97)	333,020 (19.95)	327,431 (20.32)	386,462 (22.03)	318,536 (25.69)	
40,000-	319,547 (18.53)	283,217 (16.97)	232,499 (14.43)	290,782 (16.58)	231,688 (18.69)	
50,000-	720,995 (41.82)	708,109 (42.43)	708,664 (43.97)	695,313 (39.64)	381,478 (30.77)	
70,000-	339,248 (19.68)	344,669 (20.65)	343,029 (21.28)	381,426 (21.75)	308,157 (24.85)	
Tier of City						<0.001
Tier I	35,130 (2.04)	30,359 (1.82)	36,677 (2.28)	56,102 (3.20)	49,858 (4.02)	
New Tier I	98,620 (5.72)	89,916 (5.39)	100,667 (6.25)	116,104 (6.62)	96,719 (7.80)	
Tier II	140,210 (8.13)	135,160 (8.10)	134,108 (8.32)	147,960 (8.44)	112,844 (9.10)	
Tier III	682,936 (39.61)	670,257 (40.16)	648,702 (40.25)	689,582 (39.32)	490,974 (39.60)	
Tier IV	516,935 (29.98)	512,303 (30.69)	473,324 (29.37)	504,166 (28.74)	292,385 (23.58)	
Tier V	250,309 (14.52)	231,020 (13.84)	218,145 (13.54)	240,069 (13.69)	197,079 (15.90)	
Region						<0.001
Northeast	20,090 (1.17)	16,195 (0.97)	15,965 (0.99)	17,251 (0.98)	16,320 (1.32)	
North	126,775 (7.35)	99,636 (5.97)	99,900 (6.20)	107,196 (6.11)	80,621 (6.50)	
Northwest	100,965 (5.86)	97,468 (5.84)	97,497 (6.05)	108,909 (6.21)	111,583 (9.00)	
East	425,148 (24.66)	405,644 (24.30)	373,960 (23.20)	451,801 (25.76)	339,895 (27.41)	
Central	646,124 (37.48)	652,856 (39.12)	647,695 (40.19)	618,365 (35.25)	346,283 (27.93)	
South	265,665 (15.41)	256,281 (15.36)	241,381 (14.98)	280,430 (15.99)	230,803 (18.62)	
Southwest	139,373 (8.08)	140,935 (8.44)	135,225 (8.39)	170,031 (9.69)	114,354 (9.22)	

Note: Data are presented as *n* (%).

**Table 2:** Proportions of exposure and outcomes and their relative risks across LMP years.

Year	High BMI (%)	Macrosomia (%)	LGA (%)	RR (95% CI)	
				Macrosomia	LGA
Total	849,174 (10.62)	336,701 (4.21)	691,656 (8.65)	1.40 (1.39–1.41)	1.29 (1.29–1.30)
2013	151,362 (8.78)	82,613 (4.79)	160,490 (9.31)	1.34 (1.31–1.37)	1.25 (1.23–1.27)
2014	153,023 (9.17)	74,088 (4.44)	146,860 (8.80)	1.37 (1.34–1.40)	1.27 (1.25–1.29)
2015	162,678 (10.09)	67,672 (4.20)	135,508 (8.41)	1.41 (1.38–1.44)	1.30 (1.28–1.32)
2016	222,054 (12.66)	71,112 (4.05)	148,576 (8.47)	1.46 (1.43–1.49)	1.32 (1.31–1.34)
2017	160,057 (12.91)	41,216 (3.32)	100,222 (8.08)	1.54 (1.50–1.58)	1.37 (1.35–1.39)
*p* _trend_	<0.05	<0.01	<0.05	<0.01	<0.01
APC, %	11.56 (5.36–18.13)	−7.89 [−12.40–(−3.15)]	−3.15 [−5.01–(−1.26)]	3.51 (2.39–4.64)	2.29 (1.74–2.84)

APC = annual percentage change; CI = confidence interval; LGA = large-for-gestational-age; RR = relative risk. Proportions of exposure and outcomes are presented as *n* (%). Multivariate-adjusted RRs are presented, adjusted for maternal age, paternal age, higher education, nationality, household registration, smoking, secondhand smoking, alcohol consumption, parity, infant sex, and region.

**Table 3:** Burden and Trends of Macrosomia and LGA Neonates Attributed to High Body Mass Index in China From 2013 to 2017.

Year	Macrosomia		LGA	
	Crude PAF, %	Adjusted PAF, %	Crude PAF, %	Adjusted PAF, %
Total	4.76 (4.63–4.89)	4.44 (4.33–4.54)	3.87 (3.79–3.96)	3.33 (3.26–3.40)
2013	3.54 (3.31–3.77)	3.16 (2.97–3.35)	2.82 (2.66–2.98)	2.35 (2.21–2.48)
2014	4.10 (3.85–4.36)	3.64 (3.43–3.84)	3.31 (3.14–3.49)	2.70 (2.55–2.84)
2015	4.55 (4.27–4.83)	4.32 (4.09–4.53)	3.65 (3.46–3.84)	3.20 (3.04–3.36)
2016	6.41 (6.10–6.73)	5.99 (5.74–6.23)	5.00 (4.79–5.20)	4.33 (4.15–4.50)
2017	7.40 (6.98–7.83)	7.11 (6.79–7.42)	5.59 (5.34–5.85)	5.00 (4.79–5.21)
*p* _trend_	< 0.01	< 0.01	< 0.01	< 0.01
APC, %	21.19 (13.26–29.68)	23.60 (16.76–30.85)	19.49 (12.72–26.65)	21.98 (16.14–28.11)

Crude PAFs were estimated by crude model; adjusted PAFs were estimated by multivariate-adjusted model, adjusted for maternal age, paternal age, higher education, nationality, household registration, smoking, secondhand smoking, alcohol consumption, parity, infant sex, and region. LGA = large-for-gestational-age; PAF = population attributable fraction; APC = annual percentage change.

## Data Availability

NFPCP data contained sensitive data and cannot be shared via public deposition because of information governance restrictions in place to protect individuals’ confidentiality. Access to data for external researchers (not affiliated with the National Research Institute for Family Planning) requires researchers to be physically based in the institute. Access to data is available only once approval has been obtained through the individual constituent entities controlling access to the data.

## Supplementary Materials

The following supporting information can be downloaded at: https://www.mdpi.com/article/10.3390/healthcare11030331/s1, Table S1: Table S1. Confounders-adjusted Burden and Trends of Macrosomia and LGA Neonates Attributed to High Body Mass Index in China From 2013 to 2017 After Excluding Participants With Pre-pregnancy Diabetes; Table S2. Confounders-adjusted Burden and Trends of Macrosomia and LGA Neonates Attributed to High Body Mass Index in China From 2013 to 2017 After Excluding Participants With Pre-pregnancy Hypertension; Table S3. Confounders-adjusted Burden and Trends of Macrosomia and LGA Neonates Attributed to High Body Mass Index in China From 2013 to 2017 After Excluding Participants With Pre-pregnancy Thyroid Disease; Table S4. Burden and Trends of Macrosomia and LGA Neonates Attributed to High Body Mass Index in China During 2013-2017 According to the Chinese BMI Classification Standard.

## Abbreviations

LGA	Large-for-gestational-age
BMI	Body mass index
NFPCP	National Free Pre-Pregnancy Check-ups Project
WHO	World Health Organization
LMP	Last menstrual period
RR	Relative risk
CI	Confidence interval
PAF	Population attributable fraction
APC	Annual percent change
CCDRFS	China Chronic Disease and Risk Factors Surveillance
