# Supplementary material for: Burden and Trend of Macrosomia and Large-for-Gestational-Age Neonates Attributable to High Pre-Pregnancy Body Mass Index in China, 2013–2017: A Population-Based Retrospective Cohort Study
Source: Healthcare (Basel). 2023 Jan 22;11(3):331. doi: 10.3390/healthcare11030331 (PMC9914660; doi:10.3390/healthcare11030331)
Supplement: Supplementary file 1 [file healthcare-11-00331-s001.zip › healthcare-2130173-supplementary.pdf]

healthcare

MDPI

Table S1. Confounders-adjusted Burden and Trends of Macrosomia and LGA Neonates Attributed to High Body Mass Index in China From 2013 to 2017 After Excluding Participants With Pre-pregnancy Diabetes.

Year	Macrosomia			LGA		
	**Prevalence [n (%)]**	**Multivariate-adjusted RR**	**Confounders-adjusted PAF, %**	**Prevalence [n (%)]**	**Multivariate-adjusted RR**	**Confounders-adjusted PAF, %**
Total	329 456 (4.21)	1.39 (1.37–1.40)	4.26 (4.15–4.36)	677 942 (8.66)	1.29 (1.28–1.30)	3.23 (3.16–3.30)
2013	80 770 (4.80)	1.33 (1.30–1.35)	3.03 (2.84–3.22)	157 089 (9.34)	1.24 (1.22–1.26)	2.27 (2.13–2.40)
2014	72 655 (4.45)	1.36 (1.33–1.39)	3.51 (3.30–3.72)	144 169 (8.83)	1.27 (1.25–1.28)	2.63 (2.48–2.77)
2015	66 336 (4.20)	1.40 (1.37–1.43)	4.16 (3.94–4.38)	133 039 (8.42)	1.29 (1.27–1.31)	3.11 (2.95–3.27)
2016	69 431 (4.04)	1.44 (1.41–1.47)	5.72 (5.47–5.96)	145 438 (8.46)	1.32 (1.30–1.33)	4.18 (4.01–4.36)
2017	40 264 (3.31)	1.52 (1.49–1.56)	6.82 (6.50–7.14)	98 207 (8.07)	1.36 (1.34–1.38)	4.86 (4.65–5.07)
P_{trend}	< 0.01	< 0.01	< 0.01	< 0.05	< 0.01	< 0.01
APC, %	-8.07 [-12.53–(-3.39)]	3.40 (2.31–4.50)	23.47 (17.10–30.19)	-3.29 [-5.06–(-1.49)]	2.27 (1.72–2.83)	22.04 (16.63–27.71)

Note: LAG = large-for-gestational-age; PAF = population attributable fraction; APC = annual percent change. A total of 7 832 830 participants without prepregnancy diabetes was included. Multivariate-adjusted RRs and confounders-adjusted PAFs were estimated by multivariate-adjusted model adjusted by maternal age, paternal age, higher education, nationality, household registration, smoking, secondhand smoking, alcohol consumption, parity, and region.

Table S2. Confounders-adjusted Burden and Trends of Macrosomia and LGA Neonates Attributed to High Body Mass Index in China From 2013 to 2017 After Excluding Participants With Pre-pregnancy Hypertension.

Year	Macrosomia			LGA		
	Prevalence [n (%)]	Multivariate-adjusted RR	Confounders-adjusted PAF, %	Prevalence [n (%)]	Multivariate-adjusted RR	Confounders-adjusted PAF, %
Total	328 432 (4.20)	1.39 (1.38–1.41)	4.26 (4.16–4.37)	675 496 (8.63)	1.29 (1.28–1.30)	3.22 (3.15–3.29)
2013	80 365 (4.78)	1.33 (1.30–1.36)	3.00 (2.81–3.19)	156 233 (9.29)	1.25 (1.23–1.26)	2.25 (2.11–2.38)
2014	72 402 (4.43)	1.37 (1.34–1.40)	3.52 (3.32–3.73)	143 548 (8.78)	1.27 (1.25–1.29)	2.59 (2.44–2.73)
2015	66 275 (4.19)	1.40 (1.37–1.43)	4.16 (3.94–4.38)	132 786 (8.40)	1.30 (1.28–1.32)	3.11 (2.95–3.27)
2016	69 275 (4.04)	1.45 (1.42–1.48)	5.74 (5.49–5.98)	145 085 (8.45)	1.32 (1.30–1.34)	4.18 (4.01–4.35)
2017	40 115 (3.31)	1.54 (1.50–1.58)	6.88 (6.57–7.19)	97 844 (8.06)	1.37 (1.35–1.39)	4.89 (4.68–5.10)
P_{trend}	< 0.01	< 0.01	< 0.01	< 0.05	< 0.01	< 0.01
APC, %	-7.97 [-12.49–(-3.21)]	3.56 (2.37–4.77)	23.96 (17.82–30.42)	-3.17 [-5.01–(-1.29)]	2.39 (1.72–3.05)	22.57 (16.97–28.43)

Note: LAG = large-for-gestational-age; PAF = population attributable fraction; APC = annual percent change. A total of 7 827 285 participants without prepregnancy hypertension was included.

Multivariate-adjusted RRs and confounders-adjusted PAFs were estimated by multivariate-adjusted model, adjusted by maternal age, paternal age, higher education, nationality, household registration, smoking, secondhand smoking, alcohol consumption, parity, and region.

Table S3. Confounders-adjusted Burden and Trends of Macrosomia and LGA Neonates Attributed to High Body Mass Index in China From 2013 to 2017 After Excluding Participants With Pre-pregnancy Thyroid Disease.

Year	Macrosomia			LGA		
	Prevalence [n (%)]	Multivariate-adjusted RR	Confounders-adjusted PAF, %	Prevalence [n (%)]	Multivariate-adjusted RR	Confounders-adjusted PAF, %
Total	250 967 (4.12)	1.39 (1.37–1.40)	4.24 (4.12–4.35)	520 190 (8.55)	1.29 (1.28–1.30)	3.18 (3.10–3.26)
2013	61 152 (4.76)	1.35 (1.31–1.38)	3.16 (2.94–3.38)	119 407 (9.30)	1.25 (1.23–1.27)	2.30 (2.14–2.46)
2014	55 226 (4.39)	1.36 (1.33–1.40)	3.47 (3.23–3.70)	110 040 (8.75)	1.27 (1.24–1.29)	2.57 (2.40–2.74)
2015	50 836 (4.10)	1.39 (1.36–1.42)	4.03 (3.78–4.28)	102 575 (8.27)	1.28 (1.26–1.31)	3.00 (2.82–3.18)
2016	52 856 (3.91)	1.45 (1.41–1.48)	5.71 (5.43–5.99)	112 123 (8.30)	1.31 (1.29–1.33)	4.11 (3.92–4.31)
2017	30 897 (3.23)	1.52 (1.48–1.56)	6.72 (6.35–7.08)	76 045 (7.96)	1.36 (1.34–1.39)	4.78 (4.54–5.02)
P_{trend}	< 0.01	< 0.01	< 0.01	< 0.05	< 0.01	< 0.01
APC, %	-8.51 [-12.43–(-4.42)]	3.08 (1.57–4.62)	22.24 (13.10–32.11)	-3.58 [-5.57–(-1.54)]	2.12 (1.26–2.99)	21.31 (14.06–29.02)

Note: LAG = large-for-gestational-age; PAF = population attributable fraction; APC = annual percent change. A total of 6 087 411 participants without prepregnancy thyroid disease was included. Multivariate-adjusted RRs and confounders-adjusted PAFs were estimated by multivariate-adjusted model, adjusted by maternal age, paternal age, higher education, nationality, household registration, smoking, secondhand smoking, alcohol consumption, parity, and region.

Table S4. Burden and Trends of Macrosomia and LGA Neonates Attributed to High Body Mass Index in China During 2013-2017 According to the Chinese BMI Classification Standard.

**Year**	**Macrosomia**		**LGA**	
	Crude PAF, %	Adjusted PAF, %	Crude PAF, %	Adjusted PAF, %
Total	6.40 (6.24–6.56)	5.99 (5.86–6.12)	5.33 (5.22–5.44)	4.64 (4.55–4.73)
2013	4.70 (4.41–4.99)	4.22 (3.97–4.46)	3.78 (3.59–3.98)	3.17 (3.00–3.35)
2014	5.92 (5.61–6.24)	5.34 (5.07–5.60)	5.01 (4.80–5.23)	4.21 (4.02–4.40)
2015	6.29 (5.94–6.64)	5.97 (5.69–6.25)	5.36 (5.13–5.60)	4.80 (4.60–5.00)
2016	8.24 (7.86–8.62)	7.69 (7.38–8.00)	6.41 (6.16–6.66)	5.51 (5.29–5.73)
2017	9.72 (9.21–10.24)	9.34 (8.93–9.73)	7.41 (7.10–7.72)	6.69 (6.43–6.95)
P_{trend}	< 0.01	< 0.01	< 0.01	< 0.001
APC, %	19.53 (13.54–25.83)	21.58 (17.37–25.94)	17.22 (11.19–23.59)	19.26 (13.76–25.02)

Note: Crude PAFs were estimated by crude model; adjusted PAFs were estimated by multivariate-adjusted model, adjusted for maternal age, paternal age, higher education, nationality, household registration, smoking, secondhand smoking, alcohol consumption, parity, infant sex, and region. LGA = large-for-gestational-age; PAF = population attributable fraction; APC = annual percentage change.
